# National Dental Hospital

**Published:** 1893-07-15

**Authors:** 


### FESTIVAL DINNERS AND MEETINGS.

### NATIONAL DENTAL HOSPITAL.

A meeting of subscribers to this hospital was held on Wednesday, June 28th, the Earl of Strafford in the chair. Resolutions were passed thanking the Dowager Lady Howard de Walden for her generous gift of the new buildings, which it is anticipated will be opened in October next. Subscriptions are urgently needed and will be thankfully received by the Secretary, 149, Great Portland Street, W.

